# Effect of Span 20 Feeding Zone in the Twin Screw Extruder on the Properties of Amorphous Solid Dispersion of Ritonavir

**DOI:** 10.3390/pharmaceutics15020441

**Published:** 2023-01-29

**Authors:** Hengqian Wu, Zhengping Wang, Yanna Zhao, Yan Gao, Heng Zhang, Lili Wang, Zhe Wang, Jun Han

**Affiliations:** 1 School of Chemistry and Chemical Engineering, University of Jinan, Jinan 250022, China; 2 Institute of BioPharmaceutical Research, Liaocheng University, Liaocheng 252000, China; 3 Liaocheng High-Tech Biotechnology Co., Ltd., Liaocheng 252059, China; 4 Anhui Biochem Biopharmaceutical Co., Ltd., Hefei 230088, China

**Keywords:** amorphous solid dispersion, hot-melt extrusion, ritonavir, Span 20, precipitation

## Abstract

A ternary amorphous solid dispersion (ASD) system consisting of drug/polymer/surfactant is receiving increased attention to improve the oral bioavailability of poorly water-soluble drugs. The effect of polymers has been extensively studied, while the impact of surfactants has not yet to be studied to the same extent. Challenging questions to be answered are whether the surfactants should be added with the drug or separately and the resulting differences between the two operating processes. By adjusting the liquid feeding zone for Span 20 in the hot-melt twin screw extruder equipment, we investigated the effect of Span 20 on the properties of the polyvinylpyrrolidone/vinyl acetate (PVPVA)-based ASD formulations of ritonavir. We found that with the delayed feeding positions of Span 20 in the twin screw extruder, the ability of the ternary ASDs to maintain the supersaturation of the milled extrudates was observed to be significantly enhanced. Furthermore, adding surfactant after a thorough mixing of polymer and drug could decrease the molecular mobility of ternary ASD formulations. In addition, the effects of Span 20 on the complex viscosity and structure of PVPVA were also investigated. The delayed addition of Span 20 could improve the complex viscosity of PVPVA, thus leading to the drug precipitation inhibition. In conclusion, the delayed addition of Span 20 in the twin screw extruder and prolonging the mixing time of the drug and polymer may be critical to the maintenance of supersaturation.

## 1. Introduction

Water solubility is an important physicochemical property since more than one-third of drugs are insoluble in water or poorly soluble in water [1]. Amorphous solid dispersions (ASDs) are one of the few strategies available for improving the oral bioavailability of poorly water-soluble drugs [2]. When ASDs dissolve, they can lead to a supersaturated state because of their high energy [3]. Meanwhile, drugs dispersed in the bulk aqueous phase surpassing their amorphous solubility could separate into in situ amorphous drug-rich nanodroplets [4]. This phenomenon is referred to as liquid–liquid phase separation (LLPS). The nanodroplets may serve as a reservoir in the supersaturated state to replenish the depleted drug concentration due to permeation [5]. Indulkar et al. also found that the number of drug-rich nanodroplets could increase the high rate of drug flux across the membrane [6]. Therefore, nanodroplets can lead to an improved performance in vivo. It has been documented that nucleation and crystal growth are prone to occur in the supersaturated solution due to their thermodynamic instability and these eventually could lead to precipitation [7]. It can be predicted that the solubility and absorption of the drug is consequently affected by the precipitation during ASD dissolution.

Polymer-based ASDs can satisfy two requirements: suspending the drug in an amorphous form and inhibiting precipitation from supersaturated states [8]. It has been reported that various pharmaceutical polymers can be used as precipitation inhibitors (PIs) in ASDs, such as PVPVA, polyvinyl alcohol (PVA), polyvinylpyrrolidone (PVP), hydroxypropyl methylcellulose (HPMC), hydroxypropyl methylcellulose acetate succinate (HPMC-AS), and Eudragit [9]. Several studies have investigated the effects of the above-mentioned polymers on the in vitro dissolution and supersaturation of ASD. Chen et al. characterized the crystallization tendency of drugs in aqueous media, drug–polymer interactions before and after moisture, supersaturation of drugs in the presence of polymers, and dissolution kinetics of polymers [10]. According to their findings, all these properties have implications for the dissolution performance of various ASDs. Kweku et al. reported that drug–polymer interactions that stabilize ASDs in the solid state are also important for maintaining supersaturation in solution [11]. In another study, Liu et al. observed that drug–polymer interactions in felodipine ASD systems are responsible for forming nanodroplets, further facilitating the rapid initial drug dissolution [12].

Ternary ASD systems consisting of drug/polymer/surfactant are receiving increasing attention from pharmaceutical formulation scientists in academia and industry. Surfactants commonly used in the pharmaceutical industry include Span, Tween, D-α-tocopheryl polyethylene glycol succinate (TPGS), Poloxamer, and sodium dodecyl sulfate (SDS). By reducing the interfacial energy barrier between the drug and the dissolution medium, surfactants increase the wettability and prevent the drug from precipitating in aqueous media [13]. In addition, when the concentration of surfactant is higher than the critical micelle concentration (CMC), the solubility of the drug is increased due to solubilization [14]. Most studies have demonstrated that these ternary systems could enhance the solubility and oral absorption of poorly water-soluble drugs [15]. Meng et al. reported that TPGS increased the dissolution rate and supersaturation of celecoxib ASDs consisting of celecoxib (20%), PVPVA, and TPGS at 20% level [16]. Solanki et al. revealed that the presence of 15% *w*/*w* surfactant provided up to 50% itraconazole release at pH 1 as compared to only 8% from ASDs with HPMCAS alone [17]. Nanaki et al. observed that ternary ASDs of aprepitant with Poloxamer 188 and Soluplus promoted the enhanced dissolution of drugs compared to binary aprepitant/Poloxamer 188 and aprepitant/Soluplus ASDs [18]. Chaudhari et al. suggested that the addition of surfactants to ASDs not only increases drug–polymer miscibility but also reduces recrystallization [19]. However, Fule et al. reported that although itraconazole by itself was miscible with Soluplus up to 40% *w*/*w*, the presence of Poloxamer drastically reduced its miscibility to <10% [20]. A few researches meanwhile reported that the addition of surfactants could strongly affect the inhibition of drug crystallization and the maintenance of supersaturation [21]. Although adsorption of polymers could inhibit crystal growth by blocking new growth unit incorporation sites [22], the presence of surfactants in the supersaturation solution usually reduces the adsorption of polymer molecules at the solid interface [23]. Chen et al. reported that SDS could negate the inhibitory effect of HPMC-AS on drug crystallization in posaconazole-rich amorphous precipitates by interacting competitively with HPMC-AS [24]. Ilevbare et al. revealed that surfactant molecules could accelerate crystal growth by weakening the interaction between the polymer and the crystalline solute surface [25]. Yao et al. suggested that the effect of surfactants on crystal growth and nucleation is attributed to their ability to enhance mobility [21].

In most studies mentioned above, ternary ASDs were generally prepared by spray-drying, solvent evaporation, freeze-drying, as well as by hot-melt extrusion (HME), etc. In comparison with other technologies, HME has multiple inherent advantages, including eliminating organic solvents, homogeneity of the mixture, and reduction of processing steps. Drug–polymer interactions could be enhanced by HME, as the amorphous nature of a drug is produced in the melt mixing at high shear [26]. In general, hot-melt extruders consist of a feed hopper, barrel, extrusion screws, torque sensors, heating and cooling systems, and dies [2]. By adjusting the modular components of the HME equipment, the processes can be tailored to achieve the desired outcomes and accommodate varying raw materials [27]. Screws play a critical role in the quality and quantity of material extruded in an extruder [28]. The kneading screw can extend the time that the material is in shear in the machine, resulting in a homogeneous distribution of the drug in the polymer [29]. In addition, the hot-melt extruder can be designed to have two separate hoppers for solid and liquid materials to allow surfactants to be fed in different screw zones of the extruder. We found that few publications investigated the effect of surfactants on the inhibition of drug crystallization by adjusting the surfactant feed zone in a hot-melt extruder and this observation motivated us to carry out the current work to achieve a systematic understanding of these effects.

Ritonavir, the poor equilibrium solubility and low membrane permeability drug [30], is a typical ASD product commercially produced by HME and contains PVPVA as the polymer and Span 20 as the surfactant [14]. Tho et al. reported that HME plays a vital role in the formation of the observed nano/micro dispersion of ASD containing ritonavir, PVPVA, Span 20, and fumed silica [31]. Indulkar et al. prepared ternary ASDs with ritonavir, PVPVA, and surfactants (30:70:5 *w*/*w*/*w*) by rotary evaporation [32]. In compaction to SDS, Tween 80, Span 85, and TPGS, Span 20 formed the smallest and most stable-sized droplets but induced solution crystallization. Siriwannakij et al. prepared ternary ASDs with ritonavir and PVPVA in combination with poloxamer 407 or Span 20 by HME [14]. Although the ASD containing Span 20 had a lower supersaturation than that of poloxamer 407, the supersaturation was maintained during 2 h dissolution, and the particles remained relatively small within a narrow range. Therefore, it is of vital importance to investigate the impact of Span 20 on maintaining the supersaturated state for the ritonavir ASD formulations.

In this study, ritonavir was chosen as the model compound with a relatively slow crystallization tendency [33]. We investigated the effect of the Span 20 feeding zone in the hot-melt twin screw extruder on the properties of ritonavir ASD formulations. Span 20 was fed in the solid feed zone with ritonavir–PVPVA mixtures, in the liquid feed zone between the solid feed zone and the kneading zone, and in the liquid feed zone between the two kneading zones. In order to elucidate the impact of Span 20 on the crystallization of ritonavir ASD formulations during dissolution, the analysis of the precipitation behavior of the extrudates and milled extrudates was carried out. Solid state characterization of the milled extrudates and precipitates of the ritonavir ASD formulations was also investigated to correlate the observed dissolution performance with the properties of the ASD surface.

## 2. Materials and Methods

### 2.1. Materials

Ritonavir (Polymorphic Form II) was obtained from Anhui Biochem United Pharmaceutical Co., Ltd. (Hefei, China) that was used without further purification. Ritonavir Reference Standard (99.3%) was purchased from The United States Pharmacopeial Convention (North Bethesda, MD, USA). PVPVA (Kollidon^®^ VA 64) was supplied by BASF Corporation (Ludwigshafen, Germany). Sorbitan monolaurate (Span 20-LQ-(AP)) was obtained from Croda Inc. (East Yorkshire, UK). The ultrapure water was purified by the Milli-Q^®^ Advantage A10^®^ water purification system (Millipore, MA, USA). Acetonitrile of high performance liquid chromatography (HPLC) grade was obtained by Thermo Fisher Scientific (Newington, NH, USA). Methanol was provided by Adamas Pharmaceuticals, Inc. (Shanghai, China). Potassium dihydrogen phosphate and phosphoric acid were supplied from Tianjin Zhiyuan Chemical Reagent Co., Ltd. (Tianjin, China).

### 2.2. Preparation of Ritonavir ASDs

**Figure 1:**
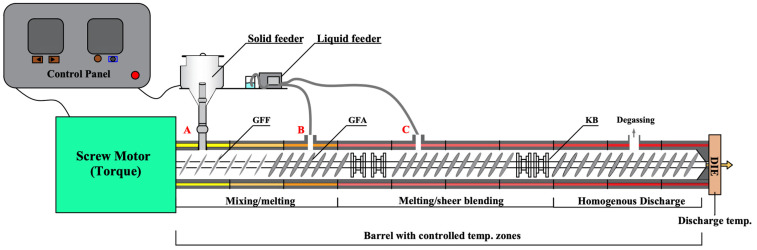
Schematic set-up of the Twin Screw Extruder for Pharma: (A) Solid feeding zone; (B) Liquid feeding zone 1; (C) Liquid feeding zone 2. GFF: conveying elements with an enlarged free volume; GFA: conveying; KB: kneading elements.

The ternary ritonavir ASDs contain PVPVA as the polymer and Span 20 as the surfactant. As shown in Figure 1, there were three different formulations due to the feeding positions of Span 20 in the hot-melt twin screw extruder for Pharma (ZSE 27 HP-PH-36D, Leistritz, Nürnberg, Germany). The screw configuration of the twin screw extruder was composed of two kneading zones and a series of conveying elements. Liquid feeding zone 1 (Figure 1B) was located between the solid feeding zone and the first kneading zone, and liquid feeding zone 2 (Figure 1C) was located between the two kneading zones. Compositions of ritonavir/PVPVA/Span 20 for all formulations were 15:75:10 *w*/*w*/*w*. Ritonavir and polymer mixtures (batch size: 5 kg each) were blended using high=shear granulators (FHSG20, Foryou Mechatronics, Shanghai, China) for 10 min to ensure the uniform mixing of materials. The stirring paddle and cutter were set at 200 rpm and 500 rpm, respectively. For Formulation A, the surfactant was slowly fed to the mixtures during the mixing process and passed to sieve #18 (1 mm opening). The powders were fed by a solid feeding zone (Figure 1A) and then extruded using a twin screw extruder in which a vacuum was applied to the extruder barrel to degas the melt. In contrast, the surfactants of Formulation B and Formulation C were fed at liquid feeding zones 1 and 2 of the twin screw extruder, respectively. The ritonavir and polymer mixtures of Formulation B and Formulation C were fed by the solid feeding zone. It is worth mentioning that the surfactant and mixtures were fed at the same rate as the prescription ratio during the HME. The total rates of powder mixtures and surfactant were 10 kg/h, and the screws were rotated at 200 rpm at a constant barrel temperature of 120 °C. The extrudate was calendered by passing through two contra-rotating calender rollers and then cooled prior to milling by a Hammer Witt FHM-67 (Frewitt, Fribourg, Switzerland) and passed through sieve #30 (600 μm opening). In addition, the physical mixtures of ritonavir/PVPVA/Span 20 were used as control.

### 2.3. Dissolution Testing

The in vitro dissolution method was established based on previous studies [34,35]. Although the use of water as a dissolution medium is discouraged because of test conditions such as the pH [36], the equilibrium solubility of ritonavir was pH-independent in non-acidic solutions [37]. In addition, the use of water as the dissolution medium also provided a more challenging environment for ritonavir precipitation, so we can better understand the supersaturated behaviors of the system. The extrudates and milled extrudates of Formulation A, Formulation B, Formulation C, and physical mixtures containing a 100 mg equivalent of ritonavir were tested for dissolution using USP dissolution apparatus II. The medium used was 900 mL of pre-degassed treated water. Dissolution testing was performed at 37 °C with a stirring speed of 75 rpm. At predetermined time points of 5, 10, 15, 20, 30, 45, 60, 90, 120, 150, 180, 210, 240, 270, and 300 min, aliquots (3 mL) were withdrawn for sampling. Methanol was added to the sampling tube to prevent any supersaturated solution from forming. The volume withdrawn from the dissolution vessel was replaced with equivalent volumes of water. All aliquots collected from the dissolution apparatus were immediately mixed with a mediated shaker. They were then filtered through 0.22 µm polytetrafluoroethylene (PTFE) syringe filters into HPLC vials for analysis. In order to analyze the phase transformation of the drug, precipitation of slurries remaining after dissolution testing was performed through a 0.45 µm polyether sulfone (PES) filter. Before further analysis, the precipitates were thoroughly dried at 60 °C for 12 h in a vacuum oven. All the dissolution testing was done in triplicates.

### 2.4. Ritonavir Analysis

Measurement of ritonavir concentrations of dissolution testing was performed using a Thermo HPLC system (Vanquish, Thermo Scientific, Waltham, MA, USA). Working standard solutions of ritonavir were prepared by suitable dilutions of standard stock solution with a mixture of methanol and water (50:50 *v*/*v*). Chromatographic separation was achieved at 25 °C using the Inertsil C18 column. The mobile phase consisted of acetonitrile and 30 mM potassium dihydrogen phosphate (55:45 *v*/*v*, pH 4.0 ± 0.1). It was degassed and filtered through a 0.45 µm membrane filter before use. The mobile phase flow rate was 1.5 mL/min, and the injection volume of each sample was 25 µL. The detective wavelength was set at 215 nm. A linear calibration curve was obtained in the 10–140 µg/mL concentration range with a regression coefficient of 0.999.

### 2.5. Particle Size Measurement

The particle size distribution of the non-filtration sample from dissolution testing at 120 min was determined using dynamic light scattering (DLS) with a Malvern Nano ZSP instrument (Malvern Instruments, Worcestershire, UK) equipped with He–Ne. The non-filtration sample from dissolution testing was added directly to the cell without further preparation. The particle size measurement procedure was initiated immediately following addition of the sample. Nanosphere^TM^ size standards (Thermo Fisher Scientific, Newington, NH, USA) were used to calibrate the instrument before the analysis.

### 2.6. Zeta Potential Determination

The zeta potential is one of the most important parameters for determining the stability of the supersaturated solution. The sample preparation for zeta potential measurements followed the same protocol as described for the particle size measurement. The values of the non-filtered sample from dissolution testing at 120 min were measured with a Zetasizer Nano Series from Malvern Instruments (Malvern Instruments, Malvern, UK) using a clear zeta potential cell at 25 °C.

### 2.7. Scanning Electron Microscopy (SEM)

The structure and morphology of physical mixtures and milled extrudates of Formulation A, Formulation B, and Formulation C were manifested by field emission SEM (Thermo Fisher Scientific FIB-SEM GX4, 5KV, Newington, NH, USA) after being placed on matrix and sputtered with gold particles. The disadvantage of this method is that it relies on the sample to be processed as a powder, so extrudates that are not milled cannot be measured. The same is true for the method below.

### 2.8. Differential Scanning Calorimetry (DSC)

The thermal properties of physical mixtures, milled extrudates of Formulation A, Formulation B, and Formulation C, and precipitate of dissolution testing were measured by differential scanning calorimeters (Discovery, TA Instruments, New Castle, DE, USA) equipped with refrigeration. Indium was used as a standard for calibrating the temperature and enthalpy. The precipitate had to be dried overnight in order to completely remove all moisture, while the milled extrudates were not further dried. We weighed 4 ± 0.5 mg of sample on an analytical balance (MSA6-6S, Sartorius, Goettingen, Germany), placed and sealed in a hermetically sealed aluminum pan with a pinhole equilibrated at 0 °C in a sample pan. To separate reversible phenomena, such as melting temperature (T_m_) and glass transition temperature (T_g_), the heating and cooling ramps of cyclic DSC curves were the heating rate of 10 °C/min from 0 °C to 180 °C, the cooling rate of 10 °C/min from 180 °C to −20 °C, then the heating rate of 10 °C/min from −20 °C to 180 °C under a dry nitrogen atmosphere. The data were analyzed with TRIOS software of TA Instruments.

### 2.9. Powder X-ray Diffraction (PXRD)

To determine physical states of physical mixtures, milled extrudates of Formulation A, Formulation B, Formulation C, and precipitates of dissolution testing, the PXRD of the samples were studied using an Ultima IV X-ray diffractometer (Rigaku, Tokyo, Japan) with CuKα radiation (1.5418 Å) at room temperature. For the preparation of samples for analysis, the scraped powder was placed on glass sample holders and then pressed to ensure a smooth and uniform surface. The diffraction pattern was measured with a tube voltage of 40 kV and a current of 40 mA. The divergence slit and anti-scattering slit were set to 0.5° for illumination of a 10 mm sample size. Scanning was performed in a 2θ range between 3° and 40° with a step size of 0.02° and a counting time of 0.1 s per step.

### 2.10. Fourier Transform Infrared (FTIR)

The intermolecular interactions among physical mixtures, milled extrudates of Formulation A, Formulation B, Formulation C, and precipitates of dissolution testing were analyzed by FTIR spectra. The spectra were recorded by a Nicolet 6700 Fourier-Transform Infrared Spectroscopy spectrometer (Thermo Scientific, Waltham, MA, USA) using the potassium bromide pellet technique within the wave number range of 4000 to 500 cm^−1^ at a resolution of 2 cm^−1^.

### 2.11. Rheological Analysis

Rheological analysis of physical mixtures and milled extrudates of Formulation A, Formulation B, and Formulation C was conducted using the MCR 302 rotational rheometer (Anton Paar, Graz, Austria). The rheometer has a diameter of 25 mm parallel plate geometry, and a zero gap was achieved between the parallel plates at the softening temperatures. After adjusting the geometry to the desired backoff distance of 5.0 cm, each sample was mounted on the hot plate at the preset temperatures and left to equilibrate for two minutes. The geometry was then used to compress the samples to 1.0 mm thickness.

Temperature sweeps were conducted at a constant frequency of 1 Hz. All samples were subjected to a strain of 1% within the linear viscoelastic region of the materials. The sample was cooled down from 150 °C to 60 °C with 2 °C/min cooling rate. Frequency sweeps were conducted at 120 °C, as the temperature was also used for HME. A constant strain of 1%, which was determined by strain amplitude tests to be in the linear viscoelastic range, was applied to all samples. Various strain frequencies varying from 100 to 0.1 rad/s were applied to the sample.

### 2.12. Near Infrared (NIR)

The NIR spectra of physical mixtures and milled extrudates of Formulation A, Formulation B, and Formulation C were measured on the NIRFlex N-500 spectrometer (Büchi, Flawil, Switzerland). Each spectrum was acquired using the NIRWare software suite. The working parameters to obtain the spectra were 32 scans at wave number range from 4000 cm^−1^ to 10,000 cm^−1^ with 4 cm^−1^ spectral resolution. The NIR measurements were conducted by rotating the samples three times and averaging the three spectra.

### 2.13. Statistical Analysis

Data analysis and graphing were undertaken using OriginPro 9.0 (OriginLab Corporation, Northampton, MA, USA). Means and standard deviations are used in expressing data.

## 3. Results and Discussion

### 3.1. Preparation of Ritonavir ASDs

As part of the present study, we aimed to develop ASD formulations of ritonavir with a drug load matching the marketed product Norvir^®^ tablets, 100 mg [38]. As a control, physical mixtures were not extruded by the twin screw extruder. Span 20 in Formulation A was fed with ritonavir–polymer mixtures. Span 20 in Formulation B was fed in the liquid feeding zone B located between the solid feeding zone and the first kneading zone. In contrast, Span 20 in Formulation C was fed in the liquid feeding zone C located between the two kneading zones. The molten ASD was mixed in the kneading zone, and, as a result, the addition of the Span 20 after the first kneading zone would enable the drug and polymer to be mixed adequately. All ritonavir ASD extrudates were transparent, light yellow, and brittle. The output critical process parameters of the twin screw extruder are shown in Table 1. Though the melt pressure and melt temperature were similar in Formulation A, Formulation B, and Formulation C, the torque and extruder load increased as the Span 20 feed position shifted back. Prior studies have noted the importance of Span 20 as a plasticizer, which has the capacity to decrease the T_g_ through a plasticizing effect [39]. Therefore, the earlier addition of Span 20 maybe increased the molecular mobility of ASD extrudates, resulting in lower torque and extruder load. Under the same milling process, milled extrudates of the three formulations had similar particle size distributions (Figure S1). The extrudates and milled extrudates were used in further investigations.

### 3.2. Physical Characterization of Ritonavir ASDs

**Figure 2:**
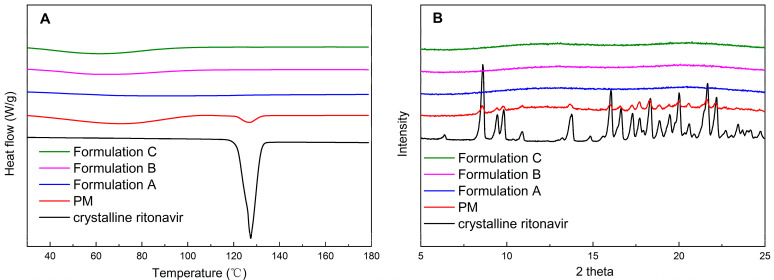
The DSC (**A**) and PXRD (**B**) of crystalline ritonavir, physical mixtures of ritonavir/PVPVA/Span 20 (PM), and milled extrudates.

Amorphous testing was performed to ensure that the milled extrudates remained physically stable in the amorphous state. DSC was utilized to elucidate the thermal transitions of crystalline ritonavir, physical mixtures, and formulations at the temperature range of 10–180 °C. As shown in Figure 2A, the melting point and endothermic peak of crystalline ritonavir for Form II were approximately 123 °C and 127 °C, respectively, corresponding to values reported previously [40]. The endothermic peak of ritonavir could also be detected in the physical mixtures of ritonavir/PVPVA/Span 20. However, DSC scans of Formulation A, Formulation B, and Formulation C showed that no crystallization of the drug was observed in ritonavir ASDs, because there was no endotherm that could be attributed to the presence of a crystalline drug.

The crystallinity of ritonavir in ASD formulations was further confirmed by PXRD. As shown in Figure 2B, the identity of the crystalline ritonavir was established by distinguishing peaks at 6.3, 8.6, 9.5, 9.8, 16.1, and 22.2 2-theta for Form II, as reported in the literature [41]. Physical mixtures of ritonavir/PVPVA/Span 20 showed intense peaks, which indicated that ritonavir exists as crystalline compounds. PXRD patterns of Formulation A, Formulation B, and Formulation C prepared by HME did not show characteristic powder diffraction peaks, which agreed with the DSC results. According to the DSC and PXRD analyses results, ritonavir in the ASD formulations was transformed from crystalline to amorphous forms during HME and remained physically stable in milling.

### 3.3. In Vitro Dissolution Studies of Ritonavir ASDs

**Figure 3:**
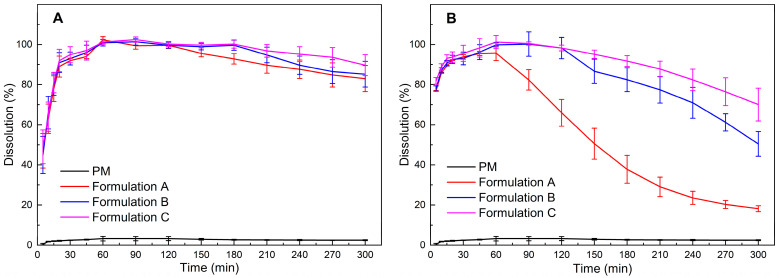
The dissolution of physical mixture of ritonavir/PVPVA/Span 20 (PM), extrudates (**A**), and milled extrudates (**B**). (mean ± S.D., *n* = 3.).

ASD formulations should be evaluated in vitro under non-sink conditions in order to determine the precipitation behavior of the drug after dispersion [9]. According to the solubility of crystalline ritonavir [5,14], the complete dissolution concentration of 100 mg ritonavir ASDs was 111.1 μg/mL in the 900 mL aqueous medium, thus indicating high supersaturation. It is only a matter of time before the occurrence of crystallization after prolonged exposure within a liquid carrier [42]. To measure the capability of extrudates and milled extrudates to maintain the supersaturation state of ritonavir, the non-sink dissolution testing was continued for 5 h in water, considering that the intestinal residence time could be up to 5 h [43]. The results of the dissolution testing of extrudates and milled extrudates are given in Figure 3. For the physical mixtures of ritonavir/PVPVA/Span 20, only about 3% of the drug was released after 1 h, whereas the extrudates and milled extrudates of three ASD formulations released >90% of the drug after 1 h. These results suggested that ritonavir was more soluble and dissolved to a greater extent because of the amorphous character of the drug in solid dispersions.

Figure 3A showed that the precipitation behavior of the three extrudates was similar. However, the precipitation rates of the milled extrudates were apparently faster relative to the unmilled extrudates (Figure 3B). The significant variables could be attributed to the crystallization of the drug, decreasing particle sizes or amorphous phase separation, probably resulting from the mechanical stress of the milling process [44]. Since Figure 2 has demonstrated that the milled extrudates still maintained the amorphous form, maybe an increase in surface area due to particle size reduction led to faster dissolution of the polymeric excipients. Consequently, the drug-release rate would increase faster until it reached supersaturation, causing earlier rapid nucleation and crystallization [42]. It was also reported that drug release from ASD was controlled by the strength of the drug–polymer interaction and the level of mixing homogeneity [44]. As the mechanical stress of milling may result in the demixing of the ASD system, the amorphous phase separation was probably another reason that induced crystallization, which lead to nucleation and precipitation of the drug [45].

However, with the delayed feeding positions of Span 20 in the twin screw extruder, the ability to maintain supersaturation of the three milled extrudates was observed with significant enhancement for ritonavir ASD formulations (Figure 3B). Thus, the addition of Span 20 in the twin screw extruder was the critical factor for the precipitation behavior. Surfactants with lower hydrophilic–lipophilic balance (HLB) values (e.g., Span 20) have been reported to interfere more with the interaction between the polymeric carrier and the drug, and therefore, could not maintain the supersaturation of the ternary system more effectively than surfactants with higher HLB values (e.g., SDS and Tween 80) [46]. Nevertheless, Span 20 can generate and stabilize the nanodroplets in the ternary system relative to SDS and Tween 80 [32]. As these nanodroplets are resolvable rapidly, they can be used to maintain maximum diffusive flux across membranes [6]. Therefore, it is essential to resist amorphous phase separation and crystallization while adding Span 20. Interestingly, the ability of Formulation C to maintain supersaturation was similar to that of the milled extrudate without the addition of Span 20 (Figure S2). These results together provide an important enlightenment that adding surfactant after the polymer and drug are thoroughly mixed could reduce the influence of surfactants on weakening the interaction between the polymeric carrier and the drug.

### 3.4. Particle Size Determination and Zeta Potential

**Figure 4:**
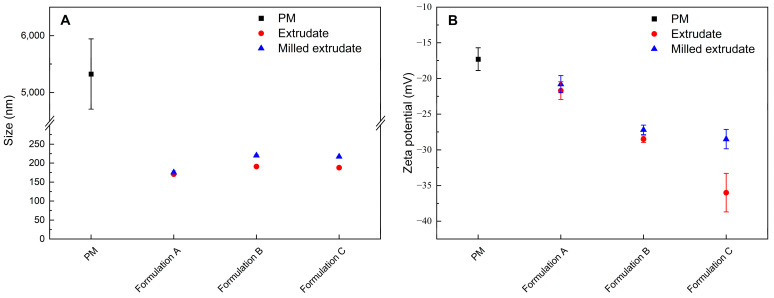
The particle size determination (**A**) and zeta potential dissolution (**B**) of the physical mixtures of ritonavir/PVPVA/Span 20 (PM), extrudates, and milled extrudates. (mean ± S.D., *n* = 3.).

To confirm whether the ASD formulations formed nanodroplets, the particle size of the non-filtration sample from dissolution testing at 1 h was measured by DLS. The results of particle size determination are given in Figure 4A. The polydispersity index (PDI) is shown in Table S1. Obviously, the physical mixture of the ritonavir/PVPVA/Span 20 without HME did not form nanodroplets. However, the size determination of the solution obtained after the dissolution of the extrudates and milled extrudates revealed the presence of nanodroplets with a size of about 200 nm (Z-average). The extrudates and milled extrudates of three ASD formulations dissolved in water were visibly turbid, which indicates the formation of light-scattering particles [31]. It was concluded that nano-dispersions were generated by dispersing melt extrudates in an aqueous medium. The results also indicated that the milling process might have no effect on the initial nanodroplets formed by the ASD formulations. 

The zeta potential of the non-filtration sample from dissolution testing at 1 h was studied. As shown in Figure 4B, the zeta potential of the physical mixture and the extrudates of Formulation A, Formulation B, and Formulation C were −17.3 mV, −21.7 mV, −28.5 mV, and −36 mV, respectively. Nanodroplets dispersions with zeta potential values of ±0–10 mV, ±10–20 mV and ±20–30 mV, and ˃±30 mV are considered highly unstable, relatively stable, moderately stable, and highly stable, respectively [47]. The results suggested that with the delayed feeding positions of Span 20 in the twin screw extruder, a significant increase in the supersaturation stability of the ASD formulations was observed. Although PVPVA is water soluble, part of the polymer may be absorbed by the nanodroplets surface, thus causing the observed negative zeta potential [31]. However, there should be no net surface charge from Span 20, since it is non-ionic. Ying et al. found that a low surfactant concentration did not affect zeta potential, and higher surfactant concentrations led to a slight decline in zeta potential [48]. Due to the surfactant concentrations in the study being much lower than the critical micelle concentration (CMC), it appeared that the delayed addition of Span 20 could improve supersaturation stability by decreasing the interaction between the surfactant weakening the polymer and the drug.

However, the zeta potentials of the milled extrudates of Formulation A, Formulation B, and Formulation C were −20.8 mV, −27.2 mV, and −28.5 mV, respectively. The trend of the zeta potential was consistent with the dissolution shown in Figure 3B. The results indicated that the milling process did not significantly affect the zeta potentials of Formulation A and Formulation B. However, Formulation C showed a significant decrease in zeta potential after milling. Interestingly, the milled extrudates of Formulation A and Formulation B precipitated significantly faster than the extrudate, but the milled extrudate of Formulation C precipitated relatively slowly. Therefore, the milled extrudates and precipitates of the three ASD formulations were further investigated.

### 3.5. Physical Characterization of Precipitates

**Figure 5:**
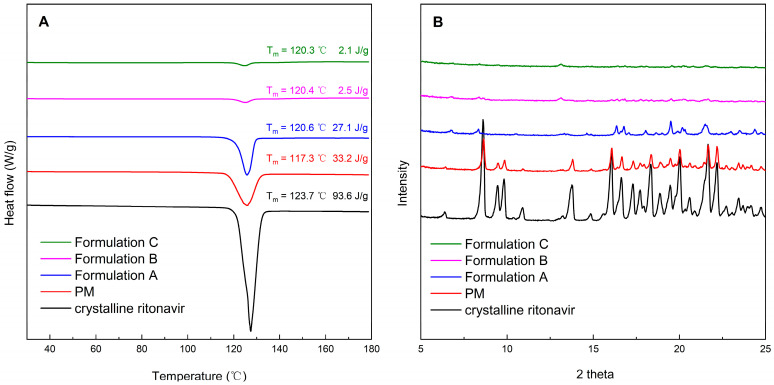
The DSC (**A**) and PXRD (**B**) of crystalline ritonavir, physical mixtures of ritonavir/PVPVA/Span 20 (PM) and precipitates of Formulation A, Formulation B, and Formulation C.

Solid state properties of the precipitates were determined using DSC and PXRD analysis at the end of dissolution testing after vacuum filtration and drying. It was observed from DSC scans in Figure 5A that precipitates obtained from dissolution testing of ASD formulations could be crystalline ritonavir, as the endothermic peak of ritonavir was detected. The results showed a decrease of crystalline ritonavir in the precipitate with the delay of the Span 20 feed position in the twin screw extruder. The trend of the endothermic peak of ASD formulations was consistent with the dissolution shown in Figure 3B. Meanwhile, Figure 5B shows PXRD patterns that support the DSC results. The precipitates obtained from dissolution testing of the physical mixture exhibited characteristic peaks of ritonavir Form II. In comparison, the precipitates of ASD formulations exhibited characteristic peaks of ritonavir Form I (Figure S3). According to the study of Barbara et al. the transformation pathway of ritonavir ASDs was amorphous > lyotropic liquid crystalline >> Form I > Form II [49]. Unless highly supersaturated solutions are present, Form I to Form II conversion is energetically unfavorable in the dosage form development [50]. Crystalline Forms I and II are very insoluble at pH 4–7, and the solubility of Form II is lower than that of Form I [37]. Moreover, the increase in supersaturation could significantly promote the rate of nucleation [51]. Under non-sink conditions, the ASD underwent crystallization to the metastable Form I. This polymorphic conversion had markedly reduced supersaturation. In addition, the area of characteristic peaks decreased gradually with the delay of the Span 20 feed position in the twin screw extruder, which was consistent with DSC. It seems that Formulation B and C had less precipitates, which exist mostly in amorphous forms with low numbers of converted crystalline forms. It has been hypothesized that drug-rich amorphous precipitates could serve as potential reservoirs of high-energy drugs, enabling enhanced and prolonged drug dissolution [24].

### 3.6. The Glass Transition Temperature of Milled Extrudates and Precipitates

**Figure 6:**
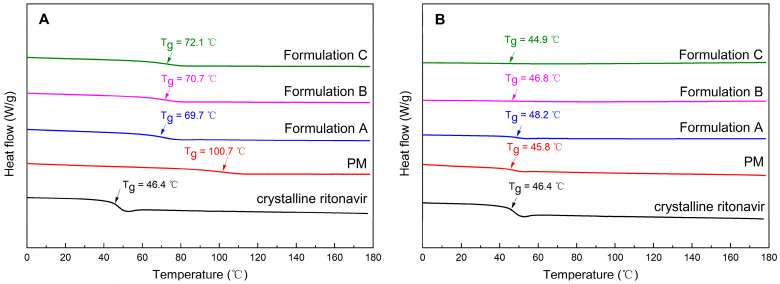
The glass transition temperature of milled extrudates (**A**) and precipitates (**B**). (PM: physical mixture of ritonavir/PVPVA/Span 20).

Given Span 20 is usually a viscous liquid at room temperature, which could accelerate crystallization and facilitate molecular mobility in ASD formulations [21]; therefore, the T_g_ values of milled extrudates and precipitates were measured. Since the heating and cooling ramps of cyclic DSC curves were applied under a dry nitrogen atmosphere, the effect of moisture content on T_g_ was also excluded as much as possible. Because the solvent evaporation method allows the drug/polymer/surfactant to exist in the solution system in a dissolved state with a higher degree of mixing [52], the T_g_ of drug/polymer/surfactant was measured using the previously described solvent evaporation method [40]. Figure S4 shows that the T_g_ of ritonavir/PVPVA/Span 20 prepared by solvent evaporation was 69.99 °C. However, the T_g_ of the physical mixture was 100.73 °C (Figure 6A), probably because it was not sufficiently mixed in the high shear granulators. The T_g_ of Formulation A after extrusion of the physical mixture was 69.74 °C, similar to the ASD prepared by solvent evaporation. The result indicated that the drug, polymer, and surfactant were well mixed in the twin screw extruder. Furthermore, the T_g_ increased gradually with the delay of the Span 20 feed position in the twin screw extruder. Ke et al. have found that different methods, such as melt-quenching, spray-drying, and ball-milling, influence the T_g_ of ASDs [53]. Molecular mobility is reduced in hot-melt blends due to a higher T_g_, resulting in physical stability [54]. This suggests that the physical stability of ASD formulations is limited by molecular mobility. In summary, these results showed that the delayed addition of Span 20 could decrease the molecular mobility of ternary ASD formulations.

As presented in Figure 6B, the T_g_ of precipitates obtained from dissolution testing of the physical mixture was 45.84 °C, similar to the T_g_ of crystalline ritonavir (Form II). The phenomenon could be attributed to the polymer and surfactant in the physical mixture dissolved in water. Contrary to the milled extrudates, the T_g_ of the precipitates decreased with the delayed addition of Span 20. Moreover, there was a tendency for the change in heat capacity during the T_g_ to become smaller. This maybe due to the fact that the precipitates contained less and less ritonavir with the delayed addition of Span 20. The result was also consistent with the dissolution in Figure 3B. In other words, the delayed addition of Span 20 had beneficial effects on the stability of the supersaturated system during dissolution and hence resulted in a better product.

### 3.7. FTIR Analysis

**Figure 7:**
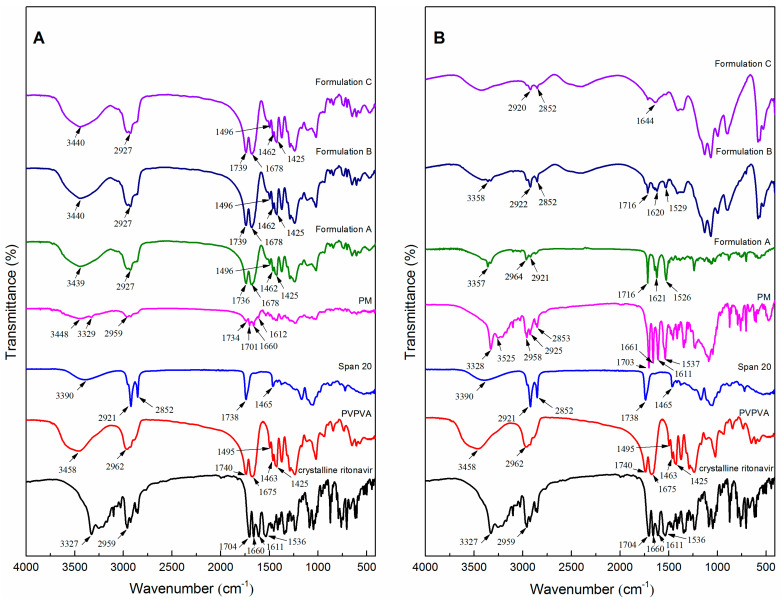
The infrared spectrum of milled extrudates (**A**) and precipitates (**B**). (PM: physical mixture of ritonavir/PVPVA/Span 20).

Intermolecular and intramolecular interactions between ritonavir and excipients in the ASD formulations were investigated by FTIR. The spectra of crystalline ritonavir (Form II), PVPVA, Span 20, and ASD formulations are given in Figure 7A. The characteristic peaks of crystalline ritonavir (Form II) at 3327 cm^−1^ (NH stretching of secondary amine), 2959 cm^−1^ (hydrogen-bonded acid within the molecules), 1704 cm^−1^ and 1660 cm^−1^ (C=O of carbamate), 1611 cm^−1^ and 1536 cm^−1^ (C=C stretching of aromatic carbons) were in agreement with the structure reported in the literature [55]. The IR spectra of Formulation A, Formulation B, and Formulation C were broadly similar, especially in the characteristic peaks representing different functional groups, which were detected in all samples. Additionally, PVPVA exhibited peaks at 1740 cm^−1^ (–C=O of the vinyl acetate) and 1675 cm^−1^ (–C=O of the vinyl pyrole), which also appeared in the spectra of the ASD formulations. Compared to the physical mixture, hydrogen bonding may be present in drug-excipients of ASD formulations. However, neither the addition of Span 20 at any position in the twin screw extruder caused any major shift in principal peaks of the ASD formulations. This may be due to the fact that the surfactants did not have much negative impact on the miscibility of the ASD formulations [14]. The spectra of the precipitates of the physical mixture and ASD formulations are presented in Figure 7B. In the precipitates of the physical mixture, the characteristic peaks of crystalline ritonavir were enhanced. The spectrum of the precipitates of ASD formulations showed distinct peaks at 1716, 1644, 1622, and 1526 cm^−1^ which correspond to peaks present in Form I of ritonavir (Figure S5), suggesting crystallization of the precipitates. The result was consistent with the spectra of PXRD shown in Figure 5B. In addition, the characteristic peak of the polymer disappeared in the precipitates of Formulation A. The result suggested that the addition of Span 20 before extrusion caused the early dissolution of the polymer.

### 3.8. The Structure and Morphology Analysis

**Figure 8:**
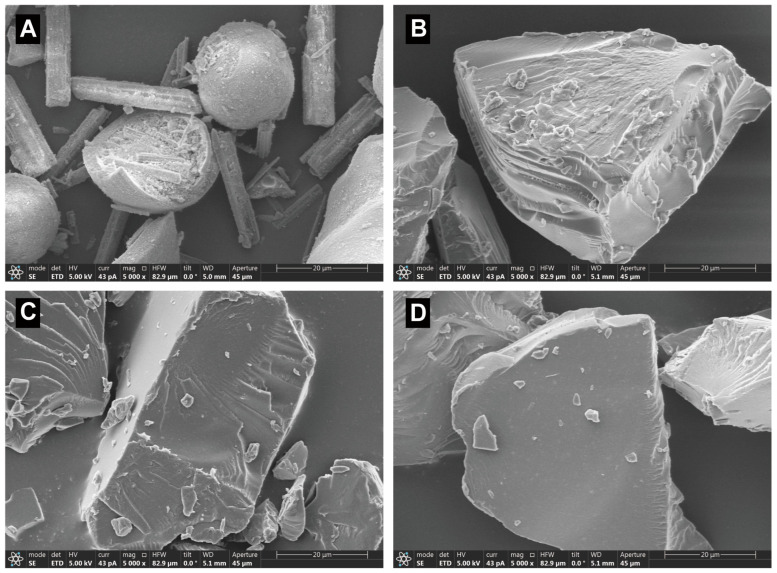
The structure and morphology of physical mixtures of ritonavir/PVPVA/Span 20 (**A**) and milled extrudate of Formulation A (**B**), Formulation B (**C**), and Formulation C (**D**).

The structure and morphology of physical mixtures and milled extrudates of Formulation A, Formulation B, and Formulation C were characterized using SEM (Figure 8). SEM analysis micrographs revealed that the physical mixtures consisted of rod-shaped drug fragments and ball-shaped PVPVA fragments (Figure 8A). It was observed that some of the drugs were encrusted into the PVPVA due to the addition of Span 20 mixing in the high shear granulators, while after the HME and milling, the shape of the milled extrudes became blocky, and the surface seemed smooth. The morphological change of the particles was a result of high temperature, shear forces, and milling. Figure 8 shows that the crystalline form disappeared to transform into amorphous forms in the milled extrudates. However, a wrinkled structure was observed on the surface of milled extrudates. The wrinkled structure decreased gradually with the delay of the Span 20 feed position in the twin screw extruder. Although the particle size distribution of these three milled extrudates was consistent, the more wrinkled structures may lead to higher surface energy and, consequently, lower physical stability [54]. This may be caused by the different viscosities of the milled extrudes of Formulation A, Formulation B, and Formulation C, resulting in variable degrees of the wrinkled structure during milling. Therefore, the viscosity of the three milled extrudates was further investigated.

### 3.9. Rheological Analysis

**Figure 9:**
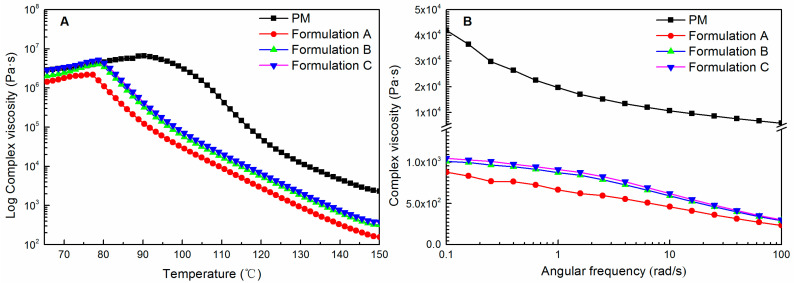
The temperature sweeps (**A**) and frequency sweeps (**B**) of the extrudates. (PM: physical mixture of ritonavir/PVPVA/Span 20).

Temperature sweeps of the physical mixtures and the three milled extrudates are depicted in Figure 9A. For the physical mixtures, the composite viscosity was highest at 90 °C and gradually decreased at higher temperatures, signifying the plasticizing effect of the dissolved ritonavir and Span 20 on PVPVA [56]. Meanwhile, no peak or bump observed for the physical mixture in complex viscosity could be attributed to the dissolution of ritonavir or melting point of the crystalline ritonavir (124 °C). This probably indicated that ritonavir was miscible with PVPVA and Span 20 at a temperature lower than its melting point, and no crystal remains at the melting point [57]. After the HME process of physical mixtures, namely Formulation A, a decrease in complex viscosity was observed across the entire temperature range. The decrease in complex viscosity followed the trend that HME was more significant than the physical mixture [57]. However, Formulation B and Formulation C were observed to have an increase in complex viscosity across the entire temperature range relative to Formulation A. In addition, the complex viscosity of Formulation C was similar to that of the physical mixture below 80 °C. The results indicated that the delayed addition of Span 20 could improve the complex viscosity of ASD formulations. Since the higher melt viscosity could generate higher torque in the extruder during processing [58], the result was consistent with the HME-measured parameters shown in Table 1.

Figure 9B displays the frequency sweeps of the physical mixtures and the three milled extrudates measured at 120 °C. Within the tested frequency region, the complex viscosity decreased significantly when the HME process was operated relative to the physical mixtures. Moreover, relative to the physical mixtures, the three milled extrudates behave more like Newtonian liquids at low frequencies [59]. However, similar to the temperature sweeps, Formulation B and Formulation C were observed to have an increase in complex viscosity in the tested frequency region relative to Formulation A. It is worth emphasizing that although the weight fraction of ritonavir was equal in all extrudates, premixing Span 20 with the drug and polymer exhibited a more plasticizing effect than the delayed addition of Span 20.

### 3.10. Near Infrared Analysis

**Figure 10:**
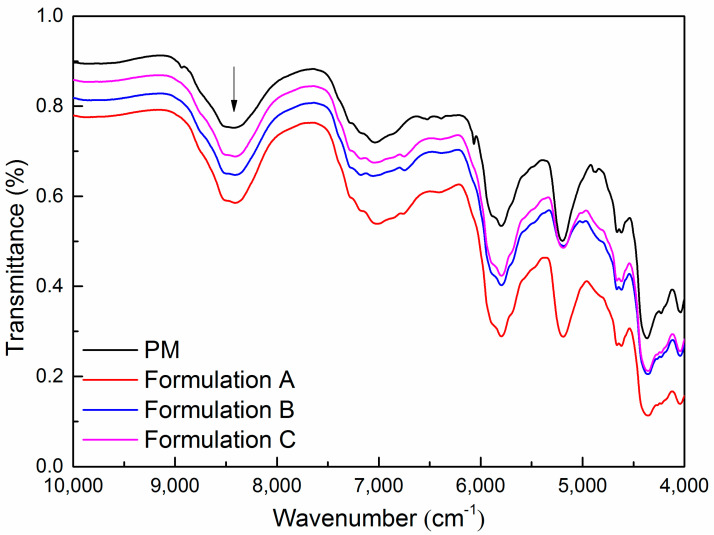
The NIR spectra of the physical mixtures and milled extrudates. (PM: physical mixture of ritonavir/PVPVA/Span 20).

So far, the discussion has emphasized the importance of the level of mixing homogeneity, molecular mobility, morphology, and polymer chain entanglements on drug precipitation inhibition of ASD formulations. Ritonavir in ASD formulations was transformed from a crystalline to amorphous form during HME and remained physically stable in milling. However, the ability to maintain supersaturation of the three milled extrudates was observed with significant enhancement for ritonavir ASD formulations with the delayed feeding positions of Span 20 in the twin screw extruder. Although there are no obvious intermolecular interactions in the three formulations, the delayed addition of Span 20 could decrease the molecular mobility of ternary ASD formulations. The variable degrees of the wrinkled structure during milling were also observed in the milled extrudates. This may be due to the delayed addition of Span 20, which could improve the complex viscosity of ASD formulations. In fact, the increase in intermolecular free volume and the accelerated mobility of polymer chains may lead to a decrease in viscosity [60]. These key factors appear to be collectively responsible for the stabilizing ability of the polymer. Since changes in polymer structure result in noticeable variations in NIR spectra [61], we investigated the effect of HME on polymer structure in the ASD formulations. The results of NIR spectra of the physical mixtures and the three milled extrudates are given in Figure 10. The spectral intensity decreased significantly when the HME process was operated relative to the physical mixtures. The substantial decrease in spectral intensity is maybe partially due to the orientation of the polymer chains towards the tensile direction [62]. It is also noted that the spectral intensity showed a gradual increase with the delayed addition of Span 20 in the twin screw extruder. Relative to the rheological analysis, the differences in spectral intensity changes will provide valuable information about the tensile deformation of polymer chains at the molecular level. 

The delayed addition of Span 20 was proven to be the optimal solution for prolonged supersaturation time. This strategy may effectively improve the bioavailability of the ternary ASD formulations. The study demonstrated that adding a surfactant after a thorough mixing of polymer and drug could decrease the molecular mobility of ternary ASD formulations. We also propose that the delayed addition of Span 20 had less interference on the ASD formation of ritonavir and PVPVA. Therefore, there was a greater amount of Span 20 acting as a surfactant/stabilizer of the nanodroplets during dissolution. In addition, the delayed addition of Span 20 could improve the complex viscosity of PVPVA, thus leading to the drug precipitation inhibition.

## 4. Conclusions

The main goal of the current study was to investigate the effect of the Span 20 feeding zone in the hot-melt twin screw extruder on the properties of ritonavir ASD formulations. Span 20 was fed in the solid feed zone with ritonavir–PVPVA mixtures, in the liquid feed zone between the solid feed zone and the kneading zone, and the liquid feed zone between the two kneading zones, respectively. According to the DSC and PXRD analyses, the ritonavir in extrudates and milled extrudates was utterly transformed from a crystal to an amorphous form during HME and remained physically stable in milling. However, the precipitation rates of the milled extrudates were apparently faster relative to the unmilled extrudates. The significant variables could be attributed to amorphous phase separation resulting from milling as the application of mechanical stress. In addition, with the delayed feeding positions of Span 20 in the twin screw extruder, the ability to maintain supersaturation of the milled extrudates was observed with a significant enhancement. These results of solid state characterization revealed that the delayed addition of Span 20 could improve supersaturation stability via reducing molecular mobility in ternary ASD formulations. Furthermore, the change in the polymer structure in the ternary ASD formulations was found to correlate with the spectral intensity of NIR. Future work could explore the validity of this correlation for the ternary ASD formulations.

## Figures and Tables

**Table 1:** Output critical process parameters of the Twin Screw Extruder.

	Torque (%)	Melt Pressure (Bar)	Melt Temperature (°C)	Extruder Load (%)
physical mixtures	/	/	/	/
Formulation A	<35	<24	<129	<49
Formulation B	<39	<23	<130	<57
Formulation C	<42	<24	<129	<59

## Data Availability

The data support the findings of this study are available on request from the corresponding author upon reasonable request.

## Supplementary Materials

The following supporting information can be downloaded at: https://www.mdpi.com/article/10.3390/pharmaceutics15020441/s1, Figure S1: The particle size distributions of milled extrudates were measured by laser diffraction measurement (Mastersizer 3000, Malvern Instruments, UK) via the dry dispersion method following standard test procedures; Figure S2: The dissolution of the ritonavir ASD formulation without the addition of Span 20 in the 900 ml aqueous medium using USP dissolution apparatus II; Figure S3: The PXRD of ritonavir Form I and Form II; Figure S4: The DSC of ritonavir ternary ASD formulations prepared by solvent evaporation; Figure S5: The infrared spectrum of ritonavir Form II; Table S1: PDI index of the particle size determination.
