# Supplementary material for: Effect of Span 20 Feeding Zone in the Twin Screw Extruder on the Properties of Amorphous Solid Dispersion of Ritonavir
Source: Pharmaceutics. 2023 Jan 29;15(2):441. doi: 10.3390/pharmaceutics15020441 (PMC9960583; doi:10.3390/pharmaceutics15020441)
Supplement: Supplementary file 1 [file pharmaceutics-15-00441-s001.zip › pharmaceutics-2128074-supplementary.pdf]

Supplemental figures:

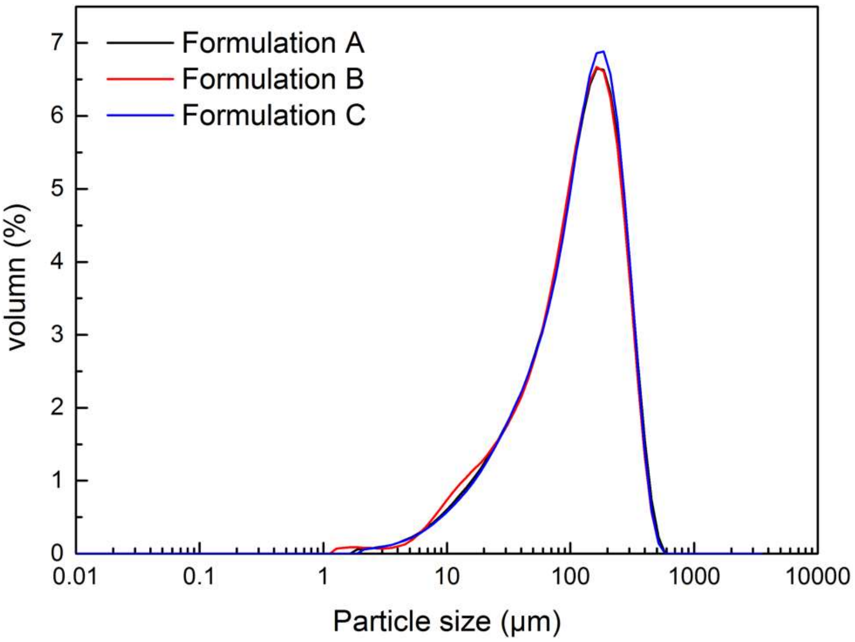

Figure S1. The particle size distributions of milled extrudates were measured by laser diffraction measurement (Mastersizer 3000, Malvern Instruments, UK) via the dry dispersion method following standard test procedures.

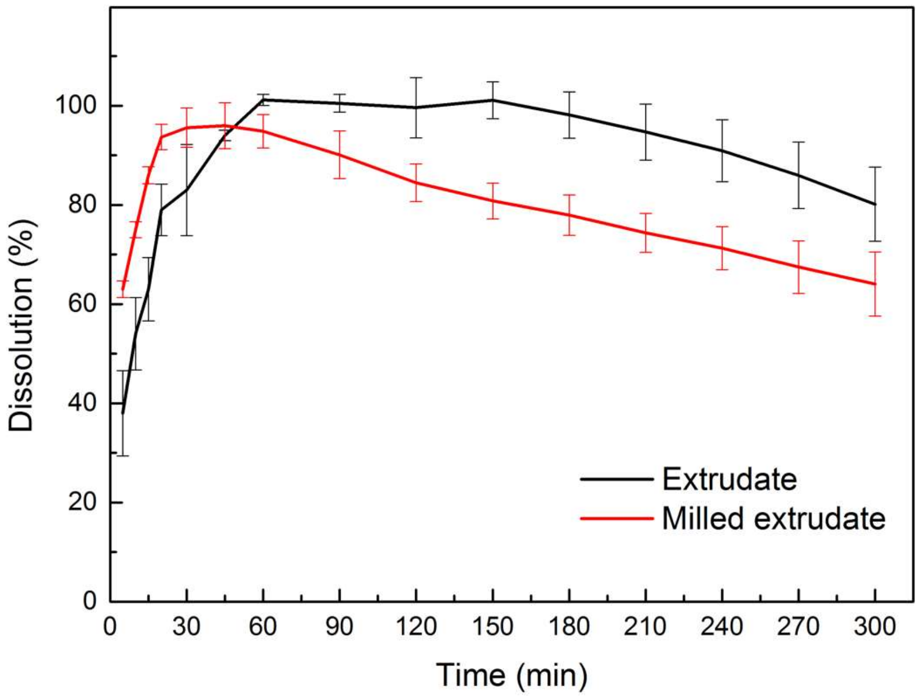

Figure S2. The dissolution of the ritonavir ASD formulation without the addition of Span 20 in the 900 ml aqueous medium using USP dissolution apparatus II.

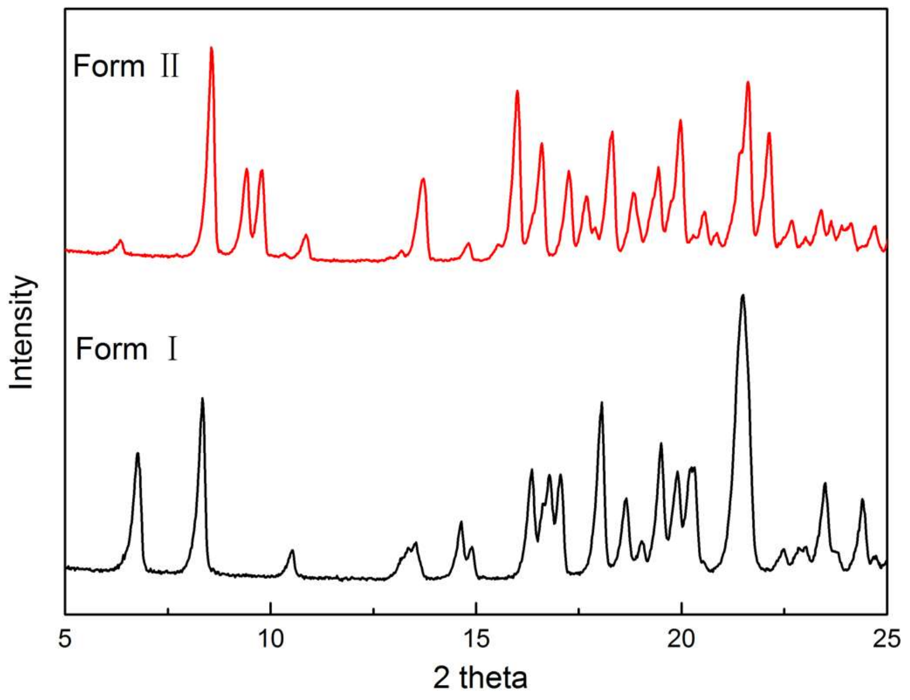

Figure S3. The PXRD of ritonavir Form I and Form II.

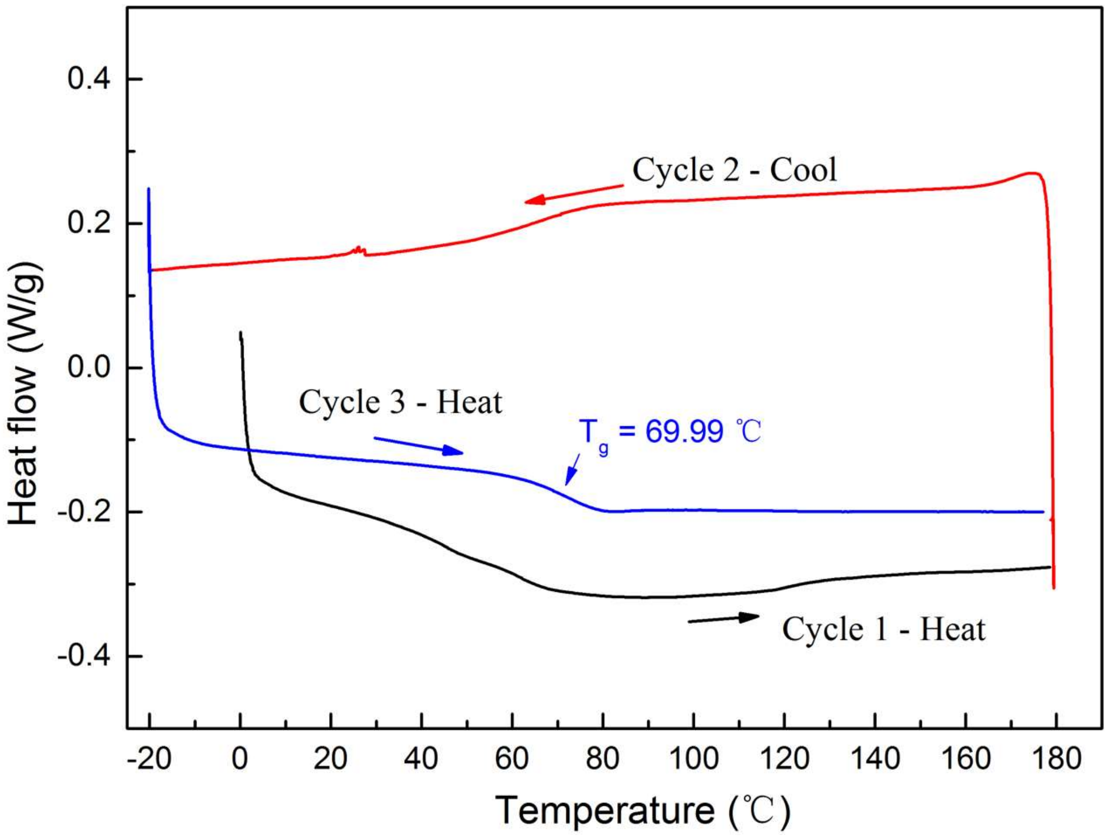

Figure S4. The DSC of ritonavir ternary ASD formulations prepared by solvent evaporation.

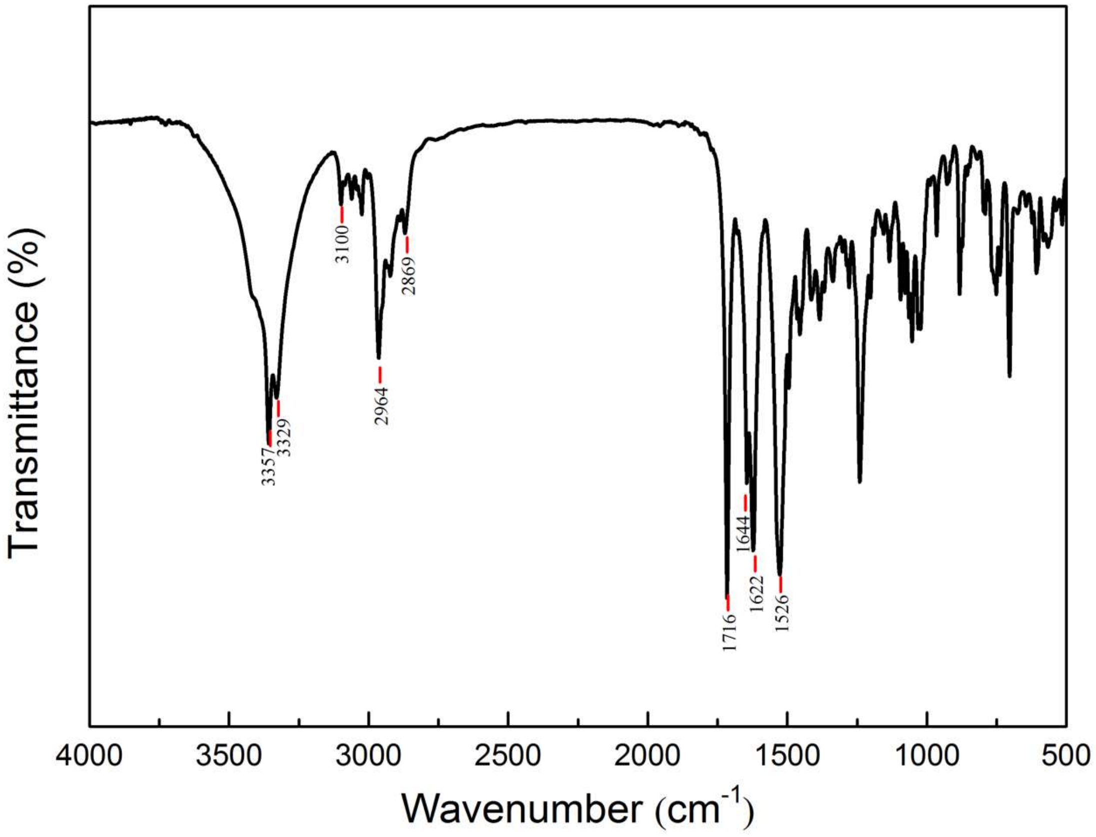

Figure S5. The infrared spectrum of ritonavir Form II.

Supplemental tables:

Table S1. PDI index of the particle size determination (mean ± SD, n = 3).

Formulations		PDI index
physical mixtures		0.93 ± 0.115
extrudates	Formulation A	0.12 ± 0.043
	Formulation B	0.12 ± 0.014
	Formulation C	0.13 ± 0.015
milled extrudates	Formulation A	0.21 ± 0.049
	Formulation B	0.17 ± 0.024
	Formulation C	0.18 ± 0.022
